# Retracted: A Study on Course of Infection and Haematological Changes in *falciparum*-Infected in Comparison with Artemisinin(s)-Treated Mice

**DOI:** 10.1155/2014/845487

**Published:** 2014-07-03

**Authors:** Malaria Research and Treatment

The article titled “A Study on Course of Infection and Haematological Changes in* falciparum*-Infected in Comparison with Artemisinin(s)-Treated Mice” [1], published in Malaria Research and Treatment, has been retracted as it was found to contain substantial flaws in its scientific content and experimental results.
